# Supplementary material for: Overlapping cell population expression profiling and regulatory inference in C. elegans
Source: BMC Genomics. 2016 Feb 29;17:159. doi: 10.1186/s12864-016-2482-z (PMC4772325; doi:10.1186/s12864-016-2482-z)
Supplement: Additional file 13: — Web supplement. (DOC 21 kb) [file 12864_2016_2482_MOESM13_ESM.zip › sortWeb/clusters/hier.300.clusters/89.html]

Cluster 89 

## Cluster 89

### Expression

| cnd-1 rep. 1 | cnd-1 rep. 2 | cnd-1 rep. 3 | pha-4 rep. 1 | pha-4 rep. 2 | pha-4 rep. 3 | ceh-27 | ceh-36 | ceh-6 | F21D5.9 | mir-57 | mls-2 | pal-1 | pros-1 | ttx-3 | unc-130 | hlh-16 | irx-1 | ceh-6 (+) hlh-16 (+) | ceh-6 (+) hlh-16 (-) | ceh-6 (-) hlh-16 (+) | cnd-1 singlets | pha-4 singlets | 0 | 60 | 120 | 150 | 180 | 240 | 330 | 390 | 420 | 480 | 540 | 570 | 600 | 630 | 660 | NAME | Functional description |
| --- | --- | --- | --- | --- | --- | --- | --- | --- | --- | --- | --- | --- | --- | --- | --- | --- | --- | --- | --- | --- | --- | --- | --- | --- | --- | --- | --- | --- | --- | --- | --- | --- | --- | --- | --- | --- | --- | --- | --- |
|  |  |  |  |  |  |  |  |  |  |  |  |  |  |  |  |  |  |  |  |  |  |  |  |  |  |  |  |  |  |  |  |  |  |  |  |  |  | ZK381.38 |  |
|  |  |  |  |  |  |  |  |  |  |  |  |  |  |  |  |  |  |  |  |  |  |  |  |  |  |  |  |  |  |  |  |  |  |  |  |  |  | *dlc-6* | Dynein Light Chain |
|  |  |  |  |  |  |  |  |  |  |  |  |  |  |  |  |  |  |  |  |  |  |  |  |  |  |  |  |  |  |  |  |  |  |  |  |  |  | F53F4.21 |  |
|  |  |  |  |  |  |  |  |  |  |  |  |  |  |  |  |  |  |  |  |  |  |  |  |  |  |  |  |  |  |  |  |  |  |  |  |  |  | C05E11.14 |  |
|  |  |  |  |  |  |  |  |  |  |  |  |  |  |  |  |  |  |  |  |  |  |  |  |  |  |  |  |  |  |  |  |  |  |  |  |  |  | C54G4.11 |  |
|  |  |  |  |  |  |  |  |  |  |  |  |  |  |  |  |  |  |  |  |  |  |  |  |  |  |  |  |  |  |  |  |  |  |  |  |  |  | T13H10.14 |  |
|  |  |  |  |  |  |  |  |  |  |  |  |  |  |  |  |  |  |  |  |  |  |  |  |  |  |  |  |  |  |  |  |  |  |  |  |  |  | *sru-33* | Serpentine Receptor, class U |
|  |  |  |  |  |  |  |  |  |  |  |  |  |  |  |  |  |  |  |  |  |  |  |  |  |  |  |  |  |  |  |  |  |  |  |  |  |  | C05B5.12 |  |
|  |  |  |  |  |  |  |  |  |  |  |  |  |  |  |  |  |  |  |  |  |  |  |  |  |  |  |  |  |  |  |  |  |  |  |  |  |  | Y43F8B.23 |  |
|  |  |  |  |  |  |  |  |  |  |  |  |  |  |  |  |  |  |  |  |  |  |  |  |  |  |  |  |  |  |  |  |  |  |  |  |  |  | R07C3.13 |  |
|  |  |  |  |  |  |  |  |  |  |  |  |  |  |  |  |  |  |  |  |  |  |  |  |  |  |  |  |  |  |  |  |  |  |  |  |  |  | C24A8.13 |  |
|  |  |  |  |  |  |  |  |  |  |  |  |  |  |  |  |  |  |  |  |  |  |  |  |  |  |  |  |  |  |  |  |  |  |  |  |  |  | F14H8.t1 |  |
|  |  |  |  |  |  |  |  |  |  |  |  |  |  |  |  |  |  |  |  |  |  |  |  |  |  |  |  |  |  |  |  |  |  |  |  |  |  | B0379.7 |  |
|  |  |  |  |  |  |  |  |  |  |  |  |  |  |  |  |  |  |  |  |  |  |  |  |  |  |  |  |  |  |  |  |  |  |  |  |  |  | *sre-32* | Serpentine Receptor, class E (epsilon) |
|  |  |  |  |  |  |  |  |  |  |  |  |  |  |  |  |  |  |  |  |  |  |  |  |  |  |  |  |  |  |  |  |  |  |  |  |  |  | W04G5.4 |  |
|  |  |  |  |  |  |  |  |  |  |  |  |  |  |  |  |  |  |  |  |  |  |  |  |  |  |  |  |  |  |  |  |  |  |  |  |  |  | *srw-3* | Serpentine Receptor, class W |
|  |  |  |  |  |  |  |  |  |  |  |  |  |  |  |  |  |  |  |  |  |  |  |  |  |  |  |  |  |  |  |  |  |  |  |  |  |  | T25B6.5 |  |

### Phenotypes enriched

none found

### Anatomy terms enriched

none found

### GO terms enriched

none found

### Expression clusters enriched

none found

### Motifs enriched

|  |  |  |  |  |  |
| --- | --- | --- | --- | --- | --- |
| **Motif** | **Logo** | **Possible orthologs** | **Number of motifs in cluster** | **Enrichment** | **FDR corrected p** |
| TFAP4\_2 |  | hlh-11 | 4 | 11.38 | 0.027 |
| MA0027.1 |  | ceh-16 | 6 | 5.66 | 0.030 |
| V$DELTAEF1\_01 |  | ztf-6 | 11 | 2.62 | 0.038 |

### Correlated (and anti-correlated) transcription factors

|  |  |
| --- | --- |
| **Transcription factor** | **Correlation** |
| efl-2 | 0.61 |
| fkh-4 | 0.58 |
| ceh-84 | 0.56 |
| tbx-37 | 0.52 |
| T26A8.4 | 0.51 |
| ztf-9 | 0.50 |
| W04B5.2 | 0.47 |
| Y48G8AL.9 | 0.47 |
| nhr-191 | 0.47 |
| hlh-34 | 0.46 |
| dpff-1 | 0.46 |
| dmd-11 | 0.44 |
| cey-1 | 0.44 |
| ceh-6 | 0.44 |
| Y116A8C.19 | 0.44 |
| ceh-89 | 0.43 |
| aptf-1 | 0.42 |
| camt-1 | 0.41 |
| hlh-14 | 0.41 |
| F57A8.1 | 0.41 |
| ZK686.5 | 0.40 |
| Y56A3A.28 | 0.40 |
| K10B3.5 | 0.40 |
| tbx-35 | 0.40 |
| nhr-73 | 0.40 |
| nhr-204 | -0.34 |
| nhr-112 | -0.34 |
| nhr-63 | -0.36 |
| ztf-1 | -0.36 |
| nhr-210 | -0.37 |
| ceh-81 | -0.38 |
| nhr-5 | -0.39 |
| nhr-121 | -0.39 |
| nhr-221 | -0.39 |
| nhr-207 | -0.41 |
| ceh-7 | -0.42 |
| pzf-1 | -0.42 |
| zip-5 | -0.43 |
| ets-9 | -0.43 |
| nhr-139 | -0.45 |
| tbx-32 | -0.45 |
| nhr-79 | -0.47 |
| nhr-232 | -0.47 |
| F55B11.4 | -0.48 |
| nhr-140 | -0.48 |
| nhr-90 | -0.50 |
| odd-1 | -0.51 |
| nhr-208 | -0.52 |
| nhr-163 | -0.52 |
| ceh-82 | -0.63 |

### ChIP peaks enriched

none found
